# Anxiety and Depression among Patients Admitted at COVID wards of East Avenue Medical Center (EAMC) Using the Validated Filipino Version of the Hospital Anxiety and Depression Scale (HADS-P)

**DOI:** 10.1192/j.eurpsy.2022.1366

**Published:** 2022-09-01

**Authors:** F.J. Guiang, M.A. Gabaon

**Affiliations:** East Avenue Medical Center, Internal Medicine, Quezon City, Philippines

**Keywords:** Depression, Anxiety, HADS, Covid-19

## Abstract

**Introduction:**

COVID-19 is a multifaceted disease establishing differences in terms of its occurrence, manner of clinical presentation and disease predilection. While mental health becomes one of the less analyzed aspects, addressing anxiety and depression allows clinicians to provide more patient-centered care in this pandemic era.

**Objectives:**

This study determined the prevalence of anxiety and depressive symptoms among patients admitted for at least 14 days at COVID wards of East Avenue Medical using the validated Filipino version of the Hospital Anxiety and Depression Scale (HADS-P), and described the socioeconomic, psychosocial, clinical factors affecting its development among COVID-19 patients.

**Methods:**

232 patients admitted at COVID ward of East Avenue Medical Center for at least 14 days were included in the study. Participants were interviewed using the validated Filipino version of the Hospital Anxiety and Depression Score (HADS-P) questionnaire. STATA 13.1 was used for data analysis at 95% confidence interval

**Results:**

Anxiety was significantly correlated with hypertension (p= 0.044), diabetes (p= 0.008), employment status (p= 0.038), and with patients who had family members with COVID- 19 (p= 0.033). Depressive symptoms occurred more likely in Chronic Kidney Disease and in COVID-19 suspects. Most participants had a normal HADS-P anxiety (6) and depression (4) median scores. Mild symptoms of anxiety (n=55) and depression (n=30) were noted among participants. The severity of COVID-19 classification was a statistically significant variable for developing anxiety symptoms.

**Conclusions:**

Identifying predictors of developing anxiety and depressive symptoms enables us to develop better strategies in addressing mental health as one of the important aspects of patient management during this pandemic.

**Disclosure:**

No significant relationships.

